# Supervised Machine-Based Learning and Computational Analysis to Reveal Unique Molecular Signatures Associated with Wound Healing and Fibrotic Outcomes to Lens Injury

**DOI:** 10.3390/ijms26157422

**Published:** 2025-08-01

**Authors:** Catherine Lalman, Kylie R. Stabler, Yimin Yang, Janice L. Walker

**Affiliations:** 1 Department of Pathology and Genomic Medicine, Thomas Jefferson University, Philadelphia, PA 19107, USA; catherine.lalman@students.jefferson.edu (C.L.); kylie.stabler@jefferson.edu (K.R.S.); 2 Sidney Kimmel Medical School, Thomas Jefferson University, Philadelphia, PA 19107, USA; 3 Department of Electrical and Computer Engineering, Western University, London, ON N6A 3K7, Canada; yimin.yang@uwo.ca; 4 Department of Ophthalmology, Thomas Jefferson University, Philadelphia, PA 19107, USA

**Keywords:** posterior capsule opacification, lens, cataract surgery, fibrosis, wound healing, repair, RNA sequencing, machine learning, biomarker discovery

## Abstract

Posterior capsule opacification (PCO), a frequent complication of cataract surgery, arises from dysregulated wound healing and fibrotic transformation of residual lens epithelial cells. While transcriptomic and machine learning (ML) approaches have elucidated fibrosis-related pathways in other tissues, the molecular divergence between regenerative and fibrotic outcomes in the lens remains unclear. Here, we used an ex vivo chick lens injury model to simulate post-surgical conditions, collecting RNA from lenses undergoing either regenerative wound healing or fibrosis between days 1–3 post-injury. Bulk RNA sequencing data were normalized, log-transformed, and subjected to univariate filtering prior to training LASSO, SVM, and RF ML models to identify discriminatory gene signatures. Each model was independently validated using a held-out test set. Distinct gene sets were identified, including fibrosis-associated genes (*VGLL3, CEBPD, MXRA7, LMNA*, gga-miR-143, RF00072) and wound-healing-associated genes (*HS3ST2, ID1*), with several achieving perfect classification. Gene Set Enrichment Analysis revealed divergent pathway activation, including extracellular matrix remodeling, DNA replication, and spliceosome associated with fibrosis. RT-PCR in independent explants confirmed key differential expression levels. These findings demonstrate the utility of supervised ML for discovering lens-specific fibrotic and regenerative gene features and nominate biomarkers for targeted intervention to mitigate PCO.

## 1. Introduction

As it is still unclear why only some cataract surgery patients develop posterior capsule opacification (PCO), many studies have sought to identify the key activators and regulators of fibrosis following injury. Complete regenerative wound healing after tissue injury is ideal but often gives way to fibrosis, a pathological scarring process driven by excessive extracellular matrix (ECM) deposition that disrupts normal tissue architecture and impairs organ function [1,2]. Fibrosis can follow diverse insults, including chronic infection, mechanical damage, inflammation, toxins, and autoimmune attack, and may affect organs such as the heart, lungs, liver, and kidneys [3,4,5,6,7]. In the lens, fibrosis is especially problematic: up to one third of adults develop PCO after cataract surgery when residual lens epithelial cells proliferate on the posterior capsule, undergo epithelial-to-mesenchymal transition and differentiate into alpha smooth muscle actin-positive (αSMA+) myofibroblasts, which persist and deposit fibrotic ECM and form an opaque layer that again obstructs vision [8,9,10,11].

Recent advances in transcriptomic profiling, including both bulk and single-cell RNA sequencing, have generated complex, high-dimensional datasets that require sophisticated analytical approaches to uncover meaningful biological insights. To address this, researchers have increasingly integrated unsupervised techniques, such as hierarchical clustering and gene co-expression network analysis, with supervised machine learning (ML) algorithms to identify robust molecular signatures associated with fibrosis. Feature selection methods including Least Absolute Shrinkage and Selection Operator (LASSO) regression, elastic net, random forest (RF) importance, support vector machine recursive feature elimination (SVM-RFE), and Boruta and XGM models have proven effective across a range of tissues and fibrotic conditions. In hepatic fibrosis, Xiong et al. applied XGBoost with SHAP feature selection to identify biomarkers of advanced fibrosis in NASH, achieving high diagnostic accuracy [12]. In addition, a machine learning-based diagnostic model for cirrhosis in chronic hepatitis B was developed, where random forest, SVM, and XGBoost models were compared, and RF was selected as the best-performing method using SHAP-based feature interpretation [13]. In idiopathic pulmonary fibrosis, Wu et al. employed WGCNA followed by LASSO, SVM-RFE, Boruta, and RF to identify three diagnostic markers from PANoptosis-related gene sets. They also identified two molecular subtypes of IPF with distinct immune infiltration profiles using consensus clustering [14]. Complementing this, Li et al. applied LASSO, RF, and SVM-RFE to bulk RNA-seq data to identify immune-related diagnostic markers that showed strong predictive power and were validated via RT-PCR in IPF tissue and TGF-β-treated epithelial cells. Their expression correlated with regulatory T-cell infiltration, highlighting immune modulation as a key feature of IPF pathogenesis [15]. In cardiac fibrosis, researchers constructed an lncRNA–miRNA–mRNA regulatory network and used random forest modeling to identify diagnostic biomarker candidates, before validating these biomarkers with RT-PCR and exploring their expression in independent datasets from heart failure patients [16]. In the kidney, a recent study by Yuan et al. employed LASSO, RF, and SVM-RFE to define a five-gene signature capable of stratifying fibrosis severity with high accuracy, which was then validated via qRT-PCR in both patient tissue and cell-based models [17].

Despite the growing number of studies applying integrated ML-based gene discovery frameworks in fibrotic disease, this approach has not yet been extended to the lens. Our study addresses this gap by combining a multi-model ML feature selection pipeline with expression-based clustering and RT-PCR validation to identify candidate genes that distinguish regenerative wound healing from fibrotic remodeling after lens injury. To investigate the molecular decision between regenerative wound healing and fibrosis in the lens, we utilized our ex vivo chick lens injury-repair model, a model that mimics surgical trauma of lens cataract surgery [18]. This model has been used among other mammalian lens models [19,20,21,22] to study regenerative and fibrotic outcomes after lens injury. Following anterior capsular incision and fiber cell removal, lenses are flattened to create an ex vivo explant to visualize the wound healing response. This system recapitulates wound healing along the fiber-cell-denuded basement membrane, which is completed by day 3, while migration off the lens capsule explant onto the stiff substrate triggers transition to alpha smooth muscle actin-positive myofibroblasts and the accumulation of a fibrotic matrix by day 3, thereby modeling the onset of lens fibrosis [23,24]. RNA-seq analysis was performed on cells undergoing wound healing vs. fibrosis in response to cataract surgery injury. In this study, we leveraged the RNA-seq dataset to identify potential molecular signatures associated with wound healing and fibrosis using several supervised machine learning models including LASSO, SVM, and RF for gene selection and classification.

## 2. Results

**Figure 1:**
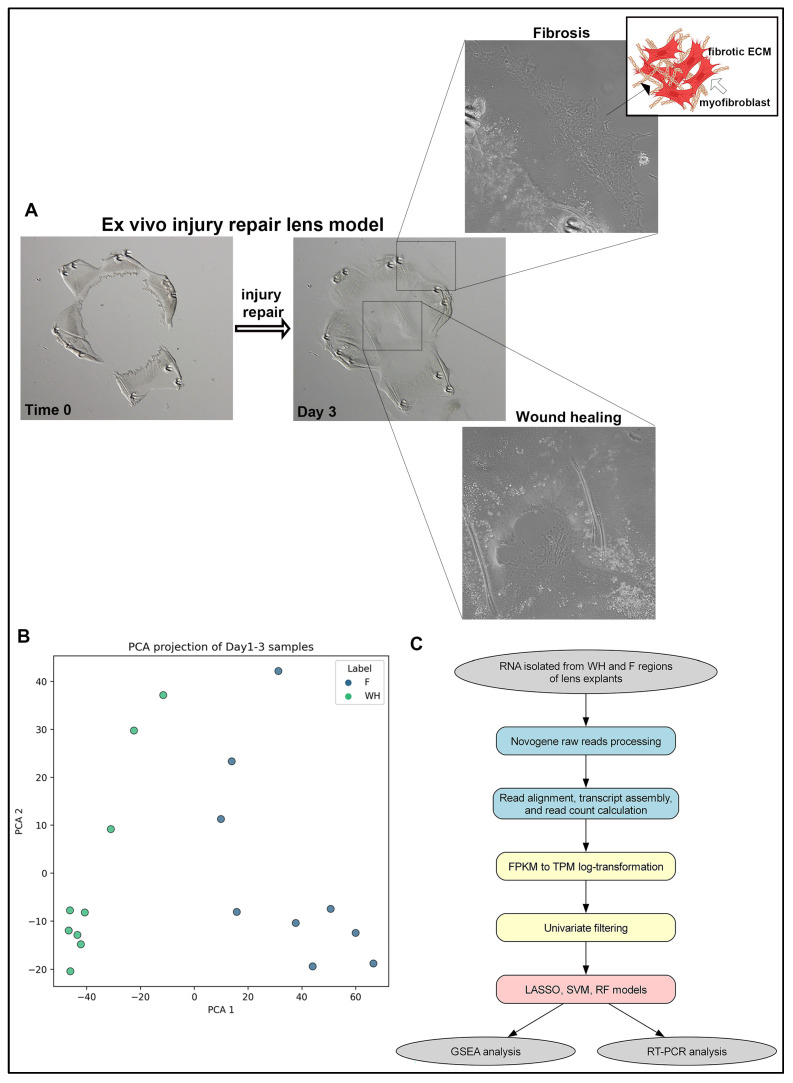
Schematic overview of the experimental and computational workflow. (**A**) Ex vivo injury-repair lens model depicted at time 0 (T0) and day 3 (D3) post-injury as wound healing is being completed across the fiber-cell-denuded basement membrane (high magnification image shown of boxed area) and cells that have moved off the lens capsule (high magnification image shown of boxed area) to initiate a fibrotic response. Cell migration off the lens capsule is shown at higher magnification, and the acquisition of the fibrotic phenotype adopted in response to injury is modeled, involving emergence of αSMA+-myofibroblast cells and accumulation of fibrotic extracellular matrix (ECM). The model was created using bioRender. (**B**) Principal component analysis shows separation of wound healing and fibrosis samples from the model. (**C**) Cartoon illustration of the pipeline integrating RNA-seq with supervised machine learning. Bulk RNA-seq was performed on lens samples collected at multiple post-injury timepoints representing wound healing (WH) and fibrosis (F) outcomes. After normalization and filtering, three machine learning models (LASSO, SVM, and random forest) were trained to distinguish WH from F. Feature selection, model validation, and biological interpretation were performed in parallel.

To characterize the molecular divergence between regenerative wound healing and fibrosis following lens injury, we utilized an established ex vivo chick lens injury-repair explant model. In this system, lenses from embryonic day 14 chick embryos were isolated, and an anterior incision was made and hydroeluted to remove fiber cells, leaving behind a monolayer of epithelial cells on the basement membrane to mimic cataract surgery. These explants were then incised and flattened epithelial side up onto tissue culture dishes to create the ex vivo lens injury capsule explants. This setup induces a spatially segregated response: cells remaining on the lens capsule undergo regenerative wound healing, while cells that migrate onto the surrounding tissue culture substrate adopt a fibrotic phenotype (Figure 1A). For transcriptomic analysis, RNA was isolated from both regions on days 1 through 3 post-injury. Principal component analysis (PCA) confirmed a clear transcriptional separation between the wound healing (WH) and fibrosis (F) samples (Figure 1B). Following normalization and log-transformation of FPKM values, a univariate filter was applied to identify genes with consistent differential expression between WH and F regions. These genes were then used to train multiple machine learning models, including LASSO, SVM, and RF, on a subset of the data, with performance being evaluated on a held-out validation set. This pipeline enabled robust identification of gene candidates with strong discriminatory power between regenerative and fibrotic responses and formed the basis for downstream GSEA and RT-PCR validation (Figure 1C).

### 2.1. Model Training and Validation Strategy for Identifying Unique Molecular Signatures Associated with Wound Healing vs. Fibrosis

**Figure 2:**
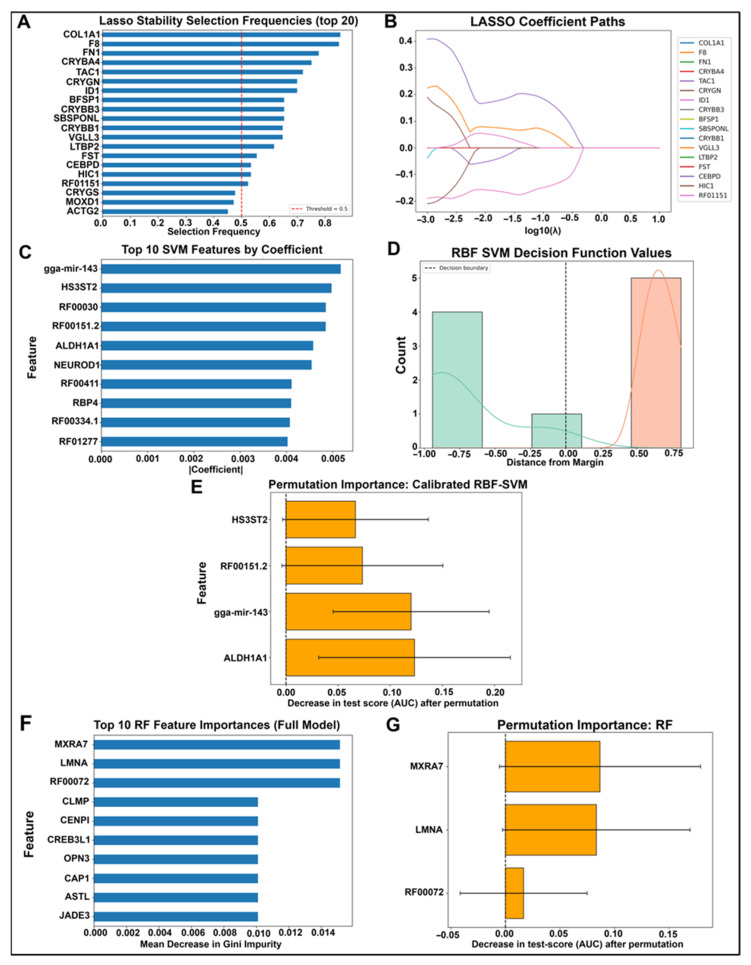
Gene importance scores across machine learning classifiers. (**A**) Top 20 genes selected by LASSO regression, ranked by absolute coefficient magnitude. (**B**) LASSO coefficient path diagram showing the evolution of gene coefficients as a function of log(λ); vertical line indicates the optimal λ chosen by cross-validation. (**C**) Top 10 linear SVM features ranked by absolute coefficient values, highlighting genes with the strongest linear decision boundary contributions. (**D**) Histogram and kernel density plot of RBF-SVM decision function values. The x-axis shows the signed distance from the SVM decision boundary (vertical dashed line at 0), with negative values predicting fibrosis (blue bars and KDE) and positive values predicting wound healing (WH) (orange bars and KDE). KDE curves represent smoothed distributions for each class, overlaid on histograms of sample counts. (**E**) Permutation importance scores for the calibrated RBF-SVM model; bars show mean ± SD of importance across permutations. (**F**) Top 10 random forest feature importances based on mean decrease in impurity. (**G**) Permutation importance scores for the random forest classifier, with genes ranked by contribution to prediction accuracy.

**Figure 3:**
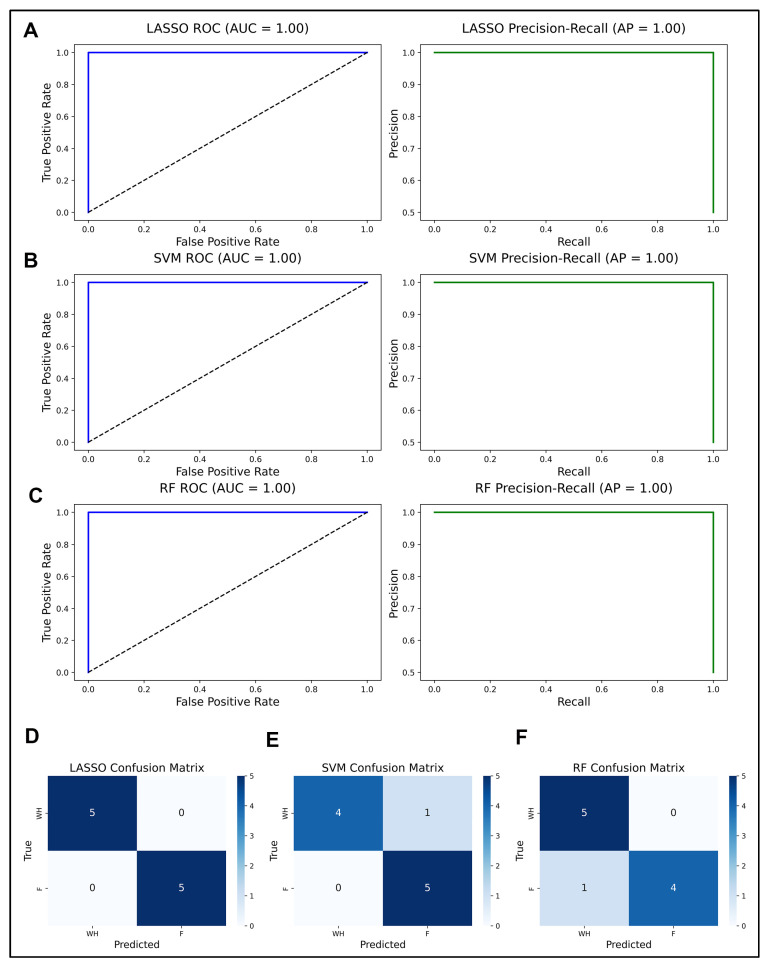
Classifier performance metrics for WH versus fibrosis prediction. (**A**–**C**) ROC and precision–recall curves for LASSO, SVM, and random forest models, respectively. (**D**–**F**) Corresponding confusion matrices for each model showing prediction accuracy, sensitivity, and specificity. Performance reflects model’s ability to distinguish regenerative from fibrotic lens states.

To identify a minimal and robust gene set capable of discriminating between fibrosis and wound healing, we applied a multistep LASSO-based feature selection and model refinement pipeline of the 13,688 genes profiled in the RNASeq dataset; 1317 passed a univariate filter based on fold change and statistical significance and were retained for downstream modeling. Stability selection using 200 bootstrap LASSO runs identified 17 genes with selection frequency ≥0.5 (Figure 2A). When these genes were refit using a three-fold cross-validated LASSO model, only three genes, which were *ID1, VGLL3*, and *CEBPD*, retained non-zero coefficients, indicating they contributed most to model performance (Figure 2B, Supplemental Figure S1). The final LASSO model trained on these three genes achieved perfect separation on the held-out validation set (AUC = 1.00, 100% accuracy; Figure 3B,D).

A linear SVM was trained on the same 1317 genes ranked features by absolute coefficient magnitude, and the top 10 are shown in Figure 2C. To evaluate their contribution, we calculated permutation importance scores for the top five genes. While gga-mir-143, *HS3ST2*, *ALDH1A1*, and RF00151.2 all showed clear drops in AUC upon permutation, RF00030 did not (Figure 2D). Therefore, RF00030 was excluded from the final feature set. A calibrated RBF-SVM trained on the remaining four genes achieved strong generalization (AUC = 1.00), correctly classifying all fibrosis samples and 80% of wound healing samples (Figure 2E and Figure 3A,B). Decision values were also clearly separated by class (Figure 2F).

Random forest modeling was then applied to the same 1317 filtered genes. Feature importance was computed from the full model using the mean decrease in Gini impurity (Figure 2G). The top 10 genes were examined further, and permutation-based AUC importance was calculated for each. Accordingly, since all three most highly ranked genes had positive permutation scores, they were retained for further analysis. The final RF model, trained on RF00072, *LMNA*, and *MXRA7*, achieved an AUC of 1.00 and correctly identified all wound healing samples, although it misclassified one fibrosis sample (Figure 3C,D).

**Figure 4:**
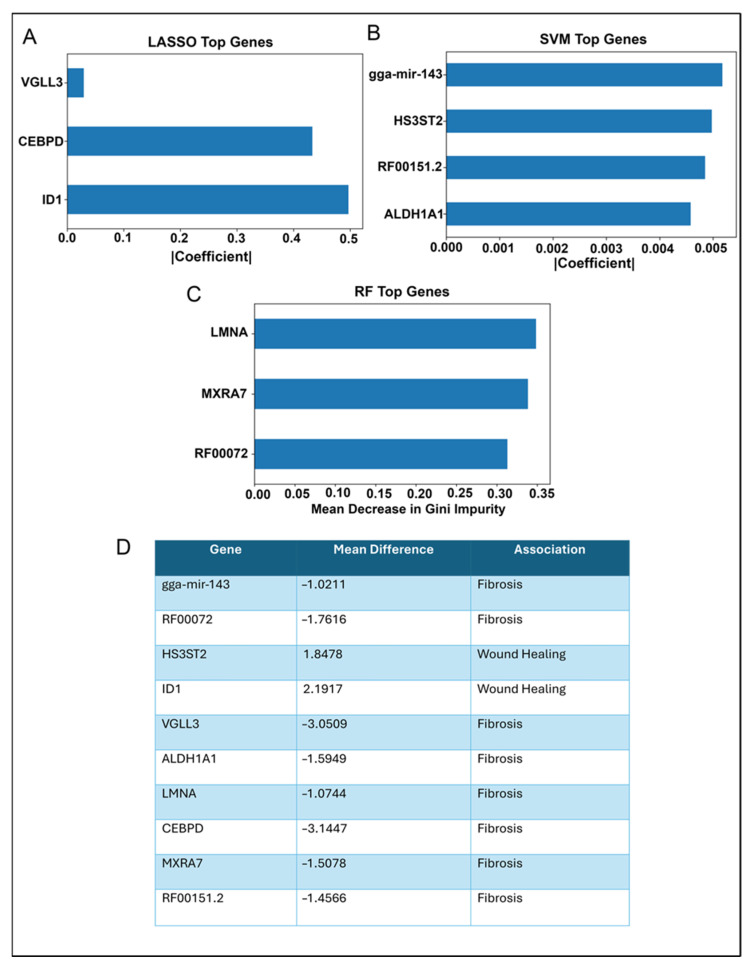
Final gene panel with model importance scores and outcome association. (**A**–**C**) Tabulated summary of the final gene set selected by at least one classifier for each machine learning tool. (**D**) Each gene’s average importance across models is listed, along with its dominant association (WH or F). This integrative panel reflects consensus features predictive of regenerative versus fibrotic responses.

Since the top genes selected by LASSO, SVM, and RF were largely non-overlapping, we retained each set independently for downstream analysis (Figure 4A–C). To assess biological relevance, we compared mean log_2_-TPM values between wound healing and fibrosis samples. Genes with positive WH-F differences were considered wound-healing-associated; those with negative differences were considered fibrosis-associated (Figure 4D).

Each model’s performance was evaluated solely on the held-out validation set. No test data were used in any stage of feature selection or training. Because hyperparameters were fixed and cross-validation confined to training data, the reported metrics provide a robust estimate of generalizability.

**Figure 5:**
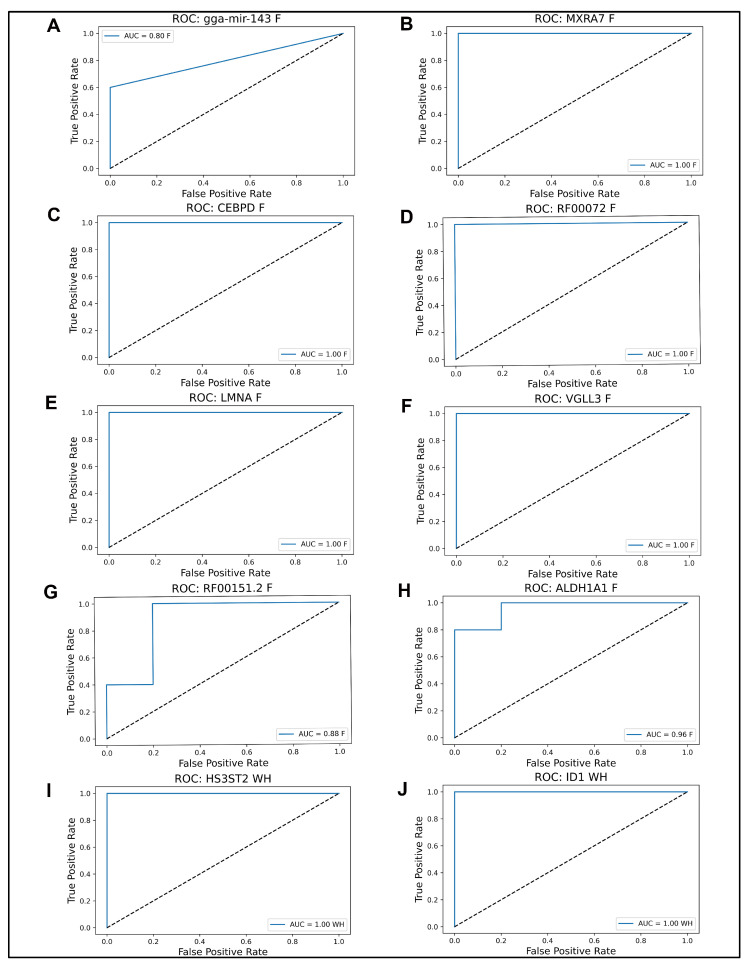
ROC curves for individual genes in the final model panel. (**A**–**J**) ROC plots showing classification performance of each gene individually in distinguishing WH from F outcomes. AUC values indicate standalone predictive power. Genes include both pro-regenerative and pro-fibrotic markers.

We next examined the consensus genes individually using ROC analysis (Figure 5A–J). Most, including *MXRA7, CEBPD, LMNA,* RF00072*, VGLL3, ID1,* and *HS3ST2,* achieved AUC = 1.00. *ALDH1A1*, RF00151.2, and gga-mir-143 reached AUCs of 0.96, 0.88, and 0.8, respectively. These results confirm that the consensus markers exhibit strong individual discriminative power and have high potential as biomarkers for wound healing and fibrosis after lens injury.

### 2.2. Validation of Potential Gene Candidates Associated with Wound Healing vs. Fibrosis

**Figure 6:**
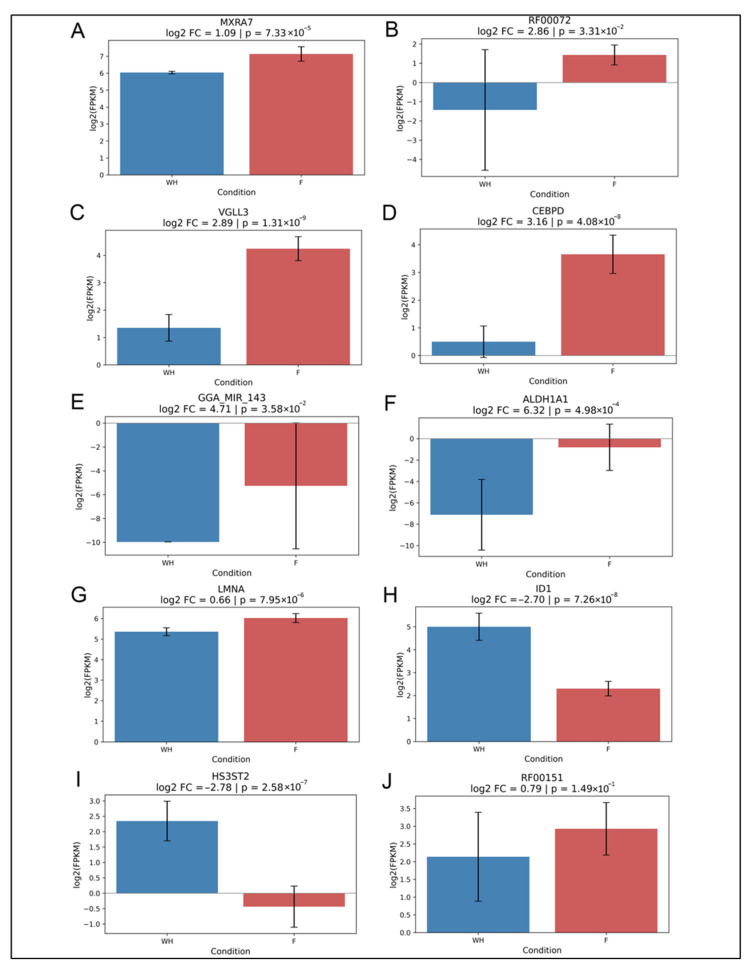
Differential expression of final model genes in WH versus fibrosis. (**A**–**J**) Bar graphs showing the log2-transformed average FPKM of pooled F samples across days D1, D2, and D3, compared to the log2-transformed average of pooled WH samples from the same time points. Statistical significance was assessed using Welch’s *t*-test; *p*-values are indicated. Color coding reflects WH or F association as inferred from model behavior.

**Figure 7:**
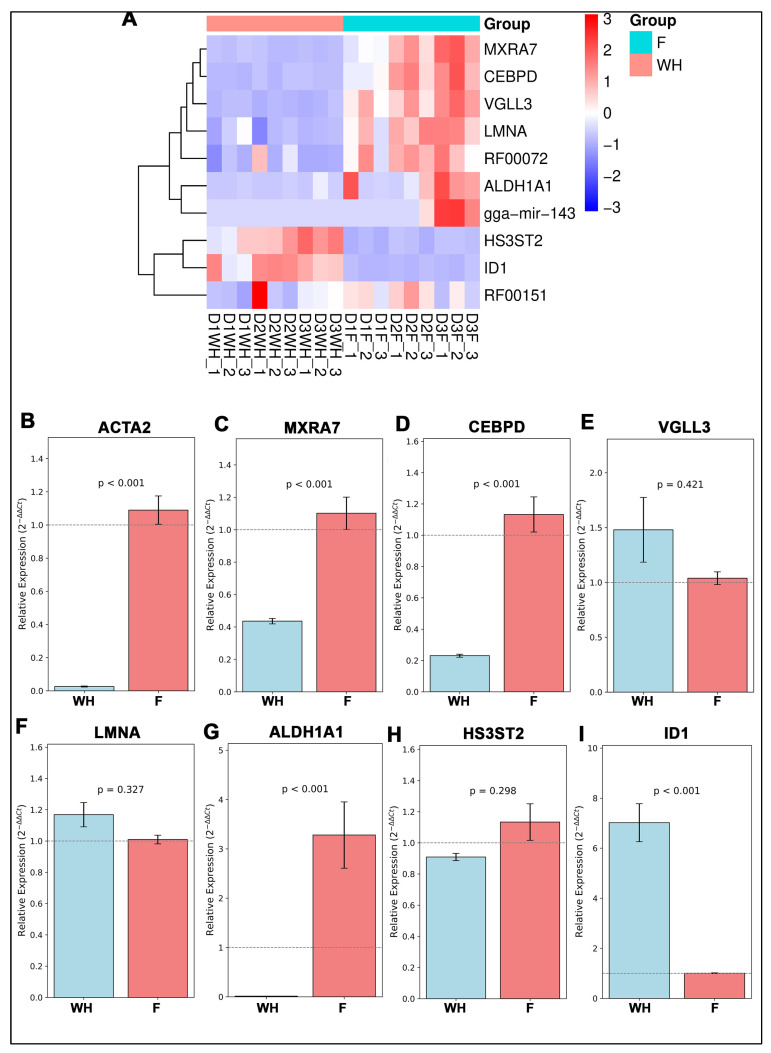
Validation of gene expression by clustered heatmap and RT-qPCR. (**A**) Heatmap showing RNA-seq expression log2-transformed FPKM of the final model genes across all samples, clustered by outcome. (**B**–**I**) RT-PCR analysis showing relative expression (2^−ΔΔCt^) for identified genes and ACTA2 to validate F vs. WH on day 3 post-injury lens explants separated into WH vs. F samples. (n = 3 individual experiments) with Welch’s *t*-test performed to calculate statistical significance.

To identify if expression of potential gene candidates was specific to wound healing vs. fibrosis, we examined the log2 fold change (log_2_FC) of gene expression for each gene candidate across pooled F and WH samples D1, D2, and D3 and created a clustered heatmap of gene expression on each individual day: D1, D2, and D3 post-injury. Expression differences for each of the 13 consensus genes were visualized using log_2_(FPKM) bar plots with 95% confidence intervals. While TPM was used for normalization and modeling, FPKM values from Novogene were used for visualization consistency. Most genes showed significant differences in expression between consolidated regenerative wound healing vs. fibrotic repair samples. For example, *MXRA7* (log_2_FC = 1.09), RF00072 (log_2_FC = 2.86), *VGLL3* (log_2_FC = 2.89), *CEBPD* (log_2_FC = 3.16), gga-mir-143 (log_2_FC = 4.71), *ALDH1A1* (log_2_FC = 6.32), and *LMNA* (log_2_FC = 0.66) showed fold changes ranging from 1.09 to 6.32 (Figure 6A–F). *ID1* (log_2_FC = −2.70) and *HS3ST2* (log_2_FC = −2.78) showed large negative log2 fold changes (Figure 6G,H). Only RF00151.2 had a modest fold change (log_2_FC = 0.79) that did not reach statistical significance (Figure 6I). Together with the clustered heatmap, our results revealed that *MXRA7, CEBPD, VGLL3, LMNA,* RF00072, *ALDH1A1,* and gga-mir-142 show preferential expression with fibrosis (Figure 6 and Figure 7A). In contrast, *HS3ST2* and *ID1* were enriched with wound healing (Figure 6 and Figure 7A). To further validate these findings, we performed RT-PCR for the seven protein-coding candidate genes utilizing Day 3 post-cataract surgery explant samples, in which RNA was isolated from cells undergoing wound healing separately from cells undergoing fibrosis. The qPCR results supported the overall trends: *MXRA7, CEBPD*, and *ALDH1A1* were significantly enriched in fibrotic samples, while *ID1* was strongly associated with wound healing. *LMNA, HS3ST2,* and *VGLL3* did not show significant expression differences. qPCR results for the fibrotic marker, *ACTA2*, indicate the specificity of the F vs. WH samples (Figure 7B). These findings reinforce the specificity of candidate gene signatures and support their utility as biomarkers for divergent post-injury outcomes in the lens (Figure 7A–H).

### 2.3. GSEA KEGG Pathway Analysis Reveals Potential Biological Function Associated with Candidate Biomarkers

**Figure 8:**
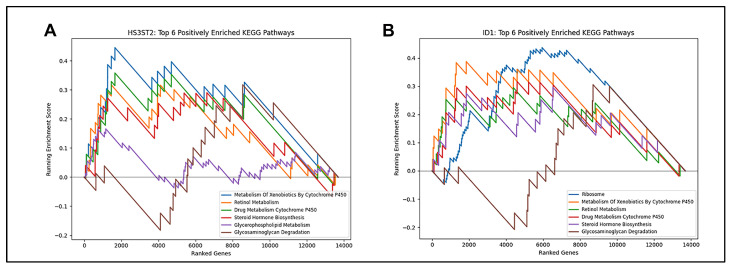
Gene Set Enrichment Analysis (GSEA) for model-selected genes associated with wound healing. (**A**,**B**) GSEA plots showing pathway-level enrichment scores for each final model gene associated with wound healing. The running enrichment score and leading-edge subsets are shown. Genes are linked to curated biological processes indicated on each enrichment score graph.

**Figure 9:**
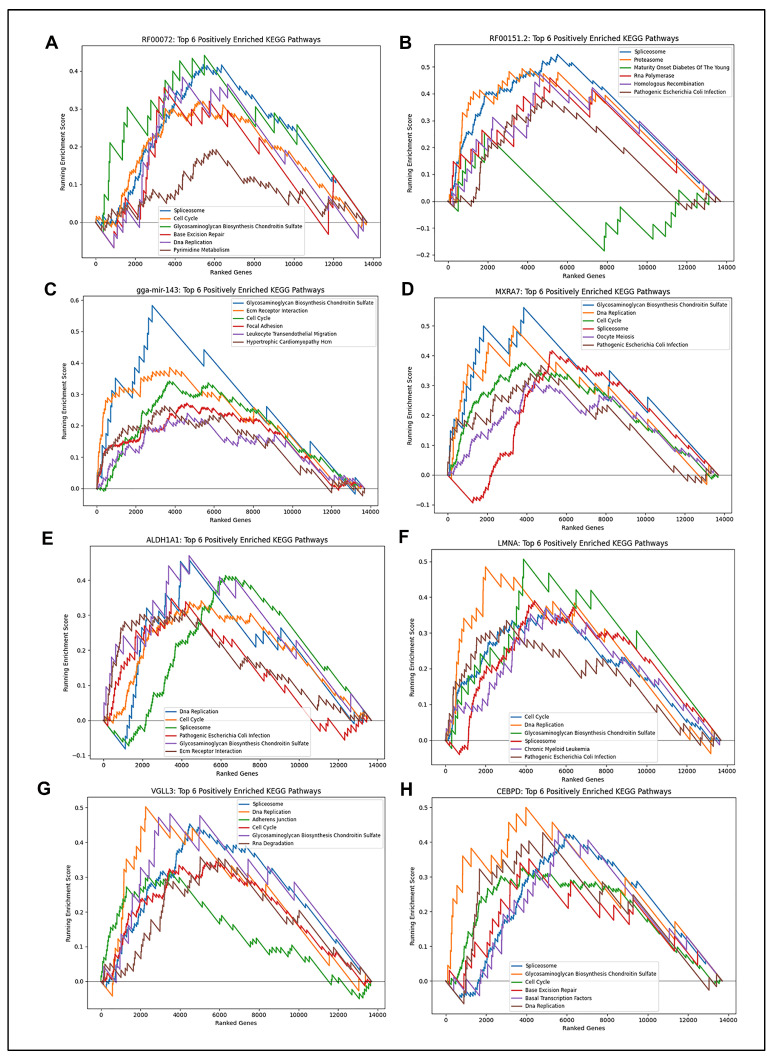
Gene Set Enrichment Analysis (GSEA) for model-selected genes associated with fibrosis. (**A**–**H**) GSEA plots showing pathway-level enrichment scores for each final model gene associated with fibrosis. The running enrichment score and leading-edge subsets are shown. Genes are linked to curated biological processes indicated on each enrichment score graph.

To evaluate the biological roles of the candidate biomarkers, Gene Set Enrichment Analysis (GSEA) was performed using KEGG pathways. Among wound-healing-associated genes, *HS3ST2* was enriched for glycosaminoglycan degradation, and *ID1* was linked to the ribosome pathway (Figure 8A,B). Among fibrosis-associated genes, gga-mir-143 and *MXRA7* were enriched for glycosaminoglycan biosynthesis, *ALDH1A1* for DNA replication, and *LMNA* for cell cycle regulation, all of which play significant roles in the synthesis of extracellular matrix and the proliferation of fibroblasts after injury (Figure 9C–F). Several of the pathways related to *VGLL3* were related to cell communication, while the majority of the pathways related to *CEBPD* were related to transcription and DNA replication (Figure 9G,H). Several others, including RF00072 and RF00151.2, were linked to the spliceosome pathway, suggesting potential coordination of post-transcriptional regulation in fibrosis (Figure 9A,B).

## 3. Discussion

PCO remains a leading complication of cataract surgery, driven by aberrant epithelial wound healing and fibrotic remodeling of the lens capsule [8]. While the molecular mechanisms distinguishing regenerative from fibrotic outcomes remain poorly defined, many studies have integrated machine learning, transcriptomic profiling, and Gene Set Enrichment Analysis to identify predictive biomarkers in other organ systems [12,13,14,15,16,17]. This study, however, is the first to apply supervised machine learning models to identify genes that may play significant roles in directing the lens’ repair response after injury. Using RNA-Seq data obtained from a chick ex vivo lens injury model capable of simulating both regenerative and fibrotic processes after mimicking cataract surgery injury, we applied LASSO, SVM, and random forest classifiers to identify candidate genes associated with wound healing or fibrosis [18]. Each model independently identified a distinct set of discriminatory genes, many of which achieved perfect classification of unseen samples. These findings provide new insight into the early transcriptional shifts that govern divergent healing outcomes and nominate several candidate regulators with high diagnostic and therapeutic potential in lens fibrosis.

Together, our ex vivo lens injury-repair model and supervised machine learning approaches identified ten genes that may direct the lens injury response toward either a regenerative wound healing or fibrotic state. GSEA analysis further revealed putative biological roles for these genes. Two, *HS3ST2* and *ID1*, were associated with wound healing. While *ID1* showed a dramatic difference in expression between the WH and F environments on D3, *HS3ST2* expression did not reach statistical significance by RT-PCR. However, *HS3ST32* did show consistent wound healing enrichment in RNA-seq data and mapped to biologically plausible pathways, including the glycosaminoglycan degradation KEGG pathway. *HS3ST2* encodes an enzyme known to selectively transfer sulfate to the 3-OH of glucosamine in heparan sulfate [25], which is subsequently exported to the ECM and conjugated to proteoglycans [26]. This modification enables heparan sulfate, and by extension *HS3ST2*, to influence diverse biological processes, including growth factor signaling [27,28,29]. Specifically, heparan sulfate can bind extracellular domains of fibroblast growth factor receptors (FGFRs), including those for FGF10, which promotes lens epithelial proliferation and fiber differentiation during development [30,31]. Additionally, heparan sulfate-bound proteoglycans can modulate TGF-β signaling, a major pro-fibrotic pathway in the lens, by promoting TβRII complex formation and increasing ligand degradation [32,33,34]. Although the role of 3-O-sulfation in the lens remains unexplored, Ferras et al. reported reduced *HS3ST1* expression in chronic kidney disease, with loss of 3-O-sulfation correlating with fibrosis severity, suggesting a protective role for *HS3ST2* in modulating growth factor availability and ECM interactions [35]. ID1, along with other ID family genes, is known to modulate TGF-β signaling via BMP activation. For example, in a study by Shu et al., BMP7 prevented TGF-β2-mediated suppression of ID2 and ID3, preserving epithelial characteristics and suggesting a pro-regenerative role for the BMP-ID axis in inhibiting cataract-associated EMT [36]. Similarly, García de Vinuesa et al. found that BMP4 suppressed TGF-β2-induced fibrosis by upregulating ID1 and ID3, which inhibited myofibroblast differentiation and ECM production [37].

Other model-identified genes function as transcriptional regulators of fibrosis. CEBPD, a member of the C/EBP family, is a well-established pro-fibrotic transcription factor that is upregulated by inflammatory cytokines such as IL-1β, IL-6, and TNF-α and in turn promotes expression of fibrosis markers including COL1A1 and fibronectin [38,39]. The upregulation of *CEBPD* was validated in several ways, first computationally using our RNASeq dataset and ML pipeline and then functionally using RT-PCR, which confirmed significantly higher *CEBPD* expression in fibrotic explants. While *CEBPD* has not been studied in the lens, it has been implicated in fibrosis in multiple organs, including the liver, heart, and kidney [39,40,41]. *VGLL3*, a transcriptional coactivator of TEAD transcription factors, acts as a mechanically responsive regulator in the Hippo pathway [42]. Increased matrix stiffness induces *VGLL3* nuclear translocation through integrin-β1 signaling and F-actin polymerization, resulting in increased synthesis of COL1A1, COL3A1, and FN1 [43]. While *VGLL3* expression did not reach statistical significance by RT-PCR, *VGLL3* does seem to play a biologically plausible role through its activity in the Hippo pathway and has been implicated in mechanotransduction and fibrosis in both the heart and liver [43,44].

Interestingly, several small nucleolar RNAs (snoRNAs), including RF00151 and RF00072, were identified as important to the fibrotic response. Although RF00151 did not show a significant *p*-value in the log_2_(FPKM) comparison, it passed the univariate filter applied prior to model training, which required a mean expression difference greater than 1 and a *p*-value less than 0.05 between WH and F groups. RF00151 is a C/D-box snoRNA involved in guiding 2′-O-methylation of 28S rRNA, while RF00072 is an H/ACA-box snoRNA guiding pseudouridylation of rRNA [45,46]. These modifications stabilize rRNA secondary structure and promote proper ribosomal folding and function [46,47]. While little is known about the role of nucleolar RNA in fibrosis, ribosome biogenesis is tightly linked to cell proliferation and growth, both of which are key features of fibrotic activation [48,49,50]. Notably, Prakash et al. showed that EMT in metastatic breast cancer is partially driven by upregulated ribosome biogenesis during G1/S arrest [51]. In parallel, *LMNA*, also identified by our model, was enriched in KEGG pathways related to DNA replication and the spliceosome. In addition to its role in nuclear architecture, lamin A regulates chromatin organization and gene expression by interacting with splicing factors such as SRSFs, which have been implicated in fibrotic responses across tissues [51,52,53]. Lamin A is also known to play a role as a “mechnostat”, responding to stiffness to alter nuclear mechanics and cell differentiation states [54,55]. Moreover, in idiopathic pulmonary fibrosis tissue, lamin A function is linked to regulating senescence in stiff environments [56]. However, *LMNA* did not show a statistically significant difference in expression between wound healing and fibrosis groups by RT-PCR, potentially reflecting post-transcriptional regulation, mechanical responsiveness, or time-dependent expression dynamics.

These findings underscore a key strength of supervised machine learning models: their ability to identify genes with predictive value based on combinatorial or synergistic expression patterns, rather than magnitude of change alone. Genes such as *LMNA*, *HS3ST2*, and *VGLL3* may contribute to class distinction not through large independent expression shifts, but through coordinated interactions with other genes that define wound healing or fibrotic states. Unlike traditional differential expression analysis, models like LASSO, SVM, and random forest leverage multivariate structure and pathway-level coherence, uncovering subtle yet biologically meaningful networks. This highlights the potential of ML-based biomarker discovery to go beyond fold-change rankings and toward mechanistic insight.

Additional genes, such as gga-miR-143 and *MXRA7*, were linked to ECM remodeling and stiffness. gga-miR-143 regulates ECM composition by repressing FSCN1, an actin-bundling cytoskeletal protein, and indirectly promoting COL3A1 expression [57,58]. Furthermore, RT-PCR confirmed significantly increased *MXRA7* expression in fibrotic samples, aligning with its predicted role in ECM remodeling. In cardiac fibrosis, gga-miR-143 targets the 3′ UTR of SPRY3 mRNA, resulting in activation of the MAPK/ERK pathway and enhanced fibroblast activation [59]. MXRA7, a matrix-interactive protein, is required for wound contraction and ECM deposition [60]. Overexpression of *MXRA7* is associated with increased collagen, α-SMA, and TGF-β expression after CCl_4_ injury in the liver, whereas knockout leads to reduced fibroblast proliferation, migration, and contractility [61,62].

Lastly, *ALDH1A1*, a detoxifying enzyme for cytotoxic aldehydes, was identified as a fibrosis-associated biomarker, and its upregulation with fibrosis was confirmed using RT-PCR. Ahadome et al. showed that ALDH1A1 supports fibroblast survival and activation by converting aldehydes like HNE to carboxylic acids, thereby reducing oxidative stress and promoting fibrotic resilience [63].

While our analysis focused on a small number of top-performing candidate genes identified through supervised machine learning, we acknowledge that many other genes showed significant expression changes after injury. These likely also hold biological relevance for lens wound healing and fibrosis. ML methods (e.g., LASSO, SVM-RFE, random forest) identify genes that best distinguish between conditions and are used to build predictive models to highlight the genes that matter most. On the other hand, enrichment tools (e.g., GO, KEGG, GSEA) interpret gene lists (differentially expressed genes) by linking them to known biological pathways and functions, and enrichment explains what those genes do. Together, ML and enrichment tools bring their own specific strengths to identify candidate factors and/or pathways that may underpin a process and/or disease development.

Interestingly, classical fibrosis-related genes such as *ACTA2, FN1*, and *COL1A1* were not among the top predictive features selected by our models. This likely reflects the fact that machine learning algorithms prioritize features based on predictive value rather than prior biological knowledge or differential expression alone. Similar findings have been reported in other ML-based fibrosis studies, where non-canonical genes often outperformed well-established markers in distinguishing disease states [14,15,17].

In conclusion, our results demonstrate the utility of combining supervised machine learning with pathway-based transcriptomic analysis to uncover early regulators of divergent healing outcomes in the lens. The gene signatures identified in this study highlight transcriptional, metabolic, and ECM-associated pathways that may serve as novel therapeutic targets for preventing PCO.

### Limitations

This study has several limitations that should be acknowledged. First, the relatively small sample size, which consists of only 18 total samples, including three independent experimental biological replicates per time point, may limit the statistical power and generalizability of the results. In addition, the limited overlap in selected genes across LASSO, SVM, and RF models likely reflects both the small sample size and the distinct feature selection strategies employed by each algorithm. In high-dimensional, low-sample-size settings, feature selection is inherently unstable; different algorithms may identify statistically equivalent but biologically distinct sets of predictors [64,65]. Despite this, all three models achieved strong predictive performance on held-out validation data, supporting the relevance of their selected features. Similar observations have been reported in other transcriptomic machine learning studies, where minimal overlap across models is common under small-sample conditions [14,15,66]. Although we applied stringent univariate filtering and used separate held-out samples for validation, further external validation using independent datasets or alternative lens fibrosis models will be necessary to confirm the robustness of the identified biomarkers. Second, while the chick ex vivo model effectively recapitulates key features of post-cataract wound healing and fibrosis, it does not fully capture the cellular and molecular complexity of human PCO, including immune interactions within the context of the entire eye and long-term tissue remodeling. Third, although the candidate genes identified by our machine learning models showed strong predictive performance and biological plausibility based on pathway enrichment, their roles in lens repair and fibrosis were inferred from transcriptomic correlations and the prior literature. We were only able to experimentally validate a subset of model-identified genes, and our RT-PCR analysis focused exclusively on protein-coding transcripts, excluding non-coding RNAs such as snoRNAs and microRNAs. Additionally, the machine learning models may prioritize genes not solely based on differential expression but on their combinatorial contribution to multigene classification patterns, meaning that some individual genes may only be predictive when analyzed in the context of larger regulatory networks. Finally, although we analyzed each machine learning model independently to preserve algorithm-specific feature selection, future integration of multi-model results and the inclusion of larger, longitudinal datasets may reveal additional shared or interacting pathways that drive fibrotic progression. Addressing these limitations in future studies will be critical for translating the identified gene signatures into therapeutic targets for preventing PCO.

## 4. Materials and Methods

### 4.1. Animals

All animal procedures were conducted in accordance with the ARVO Statement for the Use of Animals in Ophthalmic and Vision Research and were approved by the Institutional Animal Care and Use Committee (IACUC) at Thomas Jefferson University. Fertilized White Leghorn chicken eggs were purchased from Poultry Futures (Lititz, PA, USA) and incubated at 37 °C until the desired embryonic stage was reached.

### 4.2. Preparation, Imaging, and Treatment of Ex Vivo Wounded Lens Epithelial Explants

On embryonic day 14 (E14), lenses were dissected, and fiber cell masses were removed by hydroelution through a small incision in the anterior capsule. To model post-cataract surgery conditions, several additional incisions were made in the anterior epithelium, and the explants were flattened epithelial side up and pinned in 35 mm culture dishes, as previously described [18]. Explants were maintained in complete culture medium consisting of M199 (Gibco, 11150059, Waltham, MA, USA) supplemented with 10% fetal bovine serum (Gibco, A56704-02, Waltham, MA, USA), 1% L-glutamine (Corning 25-005-CI, Corning, NY, USA), and 1% penicillin–streptomycin (Corning 30-002-CI, Corning, NY, USA) at 37 °C in a humidified incubator with 5% CO_2_.

### 4.3. RNA Sequencing and Bioinformatics

For RNA-seq analysis, explants from three independent experiments were collected on days 1 through 3 after injury. Six explants were harvested per time point. RNA was isolated separately from epithelial regions that remained on the lens capsule and those that migrated into the extracapsular zone (ECZ). Lens capsules and associated cells were removed with microdissection tweezers and placed into a microcentrifuge tube containing RLT buffer, a buffer for cell lysis, prior to RNA isolation according to the manufacturer’s instructions for the RNase easy kit (Qiagen, Germantown, MD, USA). Left behind, still attached to the tissue culture dish, were cells that had migrated off the lens capsule (termed ECZ), which were scraped in RLT buffer, collected, and placed into a separate microcentrifuge tube for RNA isolation using the RNase easy kit (Qiagen). Total RNA samples were submitted to Novogene for library preparation and Illumina sequencing. Quality control steps included 1% agarose gel electrophoresis, Nanodrop spectrophotometry for RNA purity and concentration, and RNA integrity number (RIN) assessment using an Agilent 2100 Bioanalyzer. Novogene provided FPKM values, lists of differentially expressed genes (DEGs) with associated log2 fold changes, *p*-values, and adjusted *p*-values, along with preliminary bioinformatics outputs. The clustered heatmap for identified genes was created with SRplot [67]. Sequencing data are available under GEO accession number GSE298481 for samples from days 1 through 3.

For each gene expressed in D1 fibrosis (F)–D3F and D1 wound healing (WH)–D3WH samples, we first converted FPKM values to TPM to normalize for both gene length and sequencing depth, then applied a log transformation with a small offset to stabilize variance. Next, we performed a univariate filter: genes whose difference between the mean WH and F TPM values was less than 1, or whose Welch’s *p*-value exceeded 0.05 between WH and F, were removed. This left three biological replicates per time point in each condition.

### 4.4. Model Training and Validation

We randomly held out five WH and five F samples from the full dataset of 18 samples (3 WH and 3 F from each of days 1–3 post-injury) for final validation. The remaining eight samples (four WH and four F) were used for training. All model training, feature selection, and hyperparameter specification were performed exclusively on this training set. The held-out samples were used only for final model evaluation. To identify robust gene markers, we first applied an L1-penalized logistic regression (LASSO) model to the training data, which consisted of all the genes in the 4 WH and F samples that passed the filter detailed earlier. Specifically, we performed 200 bootstrap iterations using 75% subsampling without replacement and retained genes that were selected in at least 50% of iterations as “stable” features. These genes were used in a three-fold stratified cross-validated LASSO model to determine the optimal penalty parameter, which was then refit on all training data. Genes with non-zero coefficients in the final model were retained as the top LASSO-selected features. These features were used to compute ROC curves and classification metrics on the held-out test samples.

In parallel, we trained a linear support vector machine (SVM) on the same training data and ranked genes by the absolute magnitude of their coefficients. The top five genes were selected and used to train a radial basis function (RBF)-kernel SVM classifier. This calibrated model was applied to the validation set to obtain probabilistic predictions, which were used to compute ROC curves, precision–recall curves, and a confusion matrix. Permutation importance was then computed on the top five genes identified by the linear SVM and then visualized.

We also implemented a random forest (RF) approach to identify potentially significant markers. An RF classifier was trained using a random sample of 50% of the available features at each split. Feature importance values were computed from the full model using the mean decrease in Gini impurity. The top three RF-ranked genes were selected based on their high Gini importance and clear separation in internal validation and used to train a reduced-feature RF model, which was then applied to the held-out validation set for probabilistic prediction and ROC curve generation. The top RF features and their permutation importance were also visualized separately. Finally, gene-wise ROC curves were additionally computed for the retained genes from each of the LASSO, SVM, and RF models to assess individual predictive performance on the validation set. Figures were generated using Python’s matplotlib and seaborn libraries (software 3.10).

To prevent overfitting and ensure generalizability, all feature selection, parameter tuning, and model training steps were conducted exclusively on the training set. The held-out validation samples were not accessed until final model evaluation. Univariate filtering, LASSO stability selection, and gene ranking for SVM and RF models were all performed independently using only training data. Additionally, hyperparameters were fixed values based on those used in small-sample transcriptomic studies and were not tuned on the validation set. We used L1-penalized logistic regression (LASSO), support vector machines (SVM) with both linear and radial basis function (RBF) kernels, and random forest (RF) classifiers. Details of model settings, including penalty strengths and tree depth, are provided in Supplementary Table S1.

### 4.5. GSEA Analysis

To explore pathway-level associations of individual genes, we performed per-gene Gene Set Enrichment Analysis (GSEA) using the KEGG pathway database and the gseapy library in Python. Enrichment results were filtered to retain positively enriched pathways (NES > 0), and the top-ranked pathways (by FDR) were visualized using custom multi-curve enrichment plots to highlight shared co-expression trends. Enrichment results for GSEA analysis are provided in Supplementary File S1. Diagrams were generated using Python’s matplotlib and scipy libraries.

### 4.6. RT-PCR Analysis

RNA was isolated using an RNase easy kit (Qiagen, Germantown, MD, USA) from six lens injury explants in which wound healing regions were isolated separately from fibrosis regions. cDNA was created using iScript (Biorad, 1708840, Hercules, CA, USA). Three separate experiments were performed, and each sample was run in triplicate. Primers for identified gene candidates are listed in Supplementary Table S2. Gene expression was normalized to GAPDH. PCR was performed on a QuantStudio 5 Real-Time PCR instrument (Thermo Fisher, Waltham, MA, USA) using PowerUp SYBR green master mix (Applied Biosystems, A25742, Waltham, MA, USA). qPCR analysis was performed using the ΔΔCt method with fold changes calculated as 2^−ΔΔCt^ between wound healing and fibrosis samples. Graphs for each gene were generated using Python libraries matplotlib for bar plot visualization and scipy for statistical analysis. Each plot displays individual gene expression differences with error bars representing ±SEM across biological replicates and *p*-values annotated from Welch’s *t*-test comparisons.

## Data Availability

Sequencing data for days 1 through 3 have been submitted under accession number GSE298481.

## Supplementary Materials

The following supporting information can be downloaded at https://www.mdpi.com/article/10.3390/ijms26157422/s1.

## Abbreviations

The following abbreviations are used in this manuscript:

PCO	Posterior Capsule Opacification
ML	Machine Learning
LASSO	Least Absolute Shrinkage and Selection Operator
SVM	Support Vector Machine
RF	Random Forest
ECM	Extracellular Matrix
SVM-RFE	Support Vector Machine Recursive Feature Elimination
PCA	Principal Component Analysis
WH	Wound Healing
F	Fibrosis
GSEA	Gene Set Enrichment Analysis
