# Supplementary material for: Supervised Machine-Based Learning and Computational Analysis to Reveal Unique Molecular Signatures Associated with Wound Healing and Fibrotic Outcomes to Lens Injury
Source: Int J Mol Sci. 2025 Aug 1;26(15):7422. doi: 10.3390/ijms26157422 (PMC12347510; doi:10.3390/ijms26157422)
Supplement: Supplementary file 1 [file ijms-26-07422-s001.zip › Tables S1 and S2 and Figure S1.pdf]

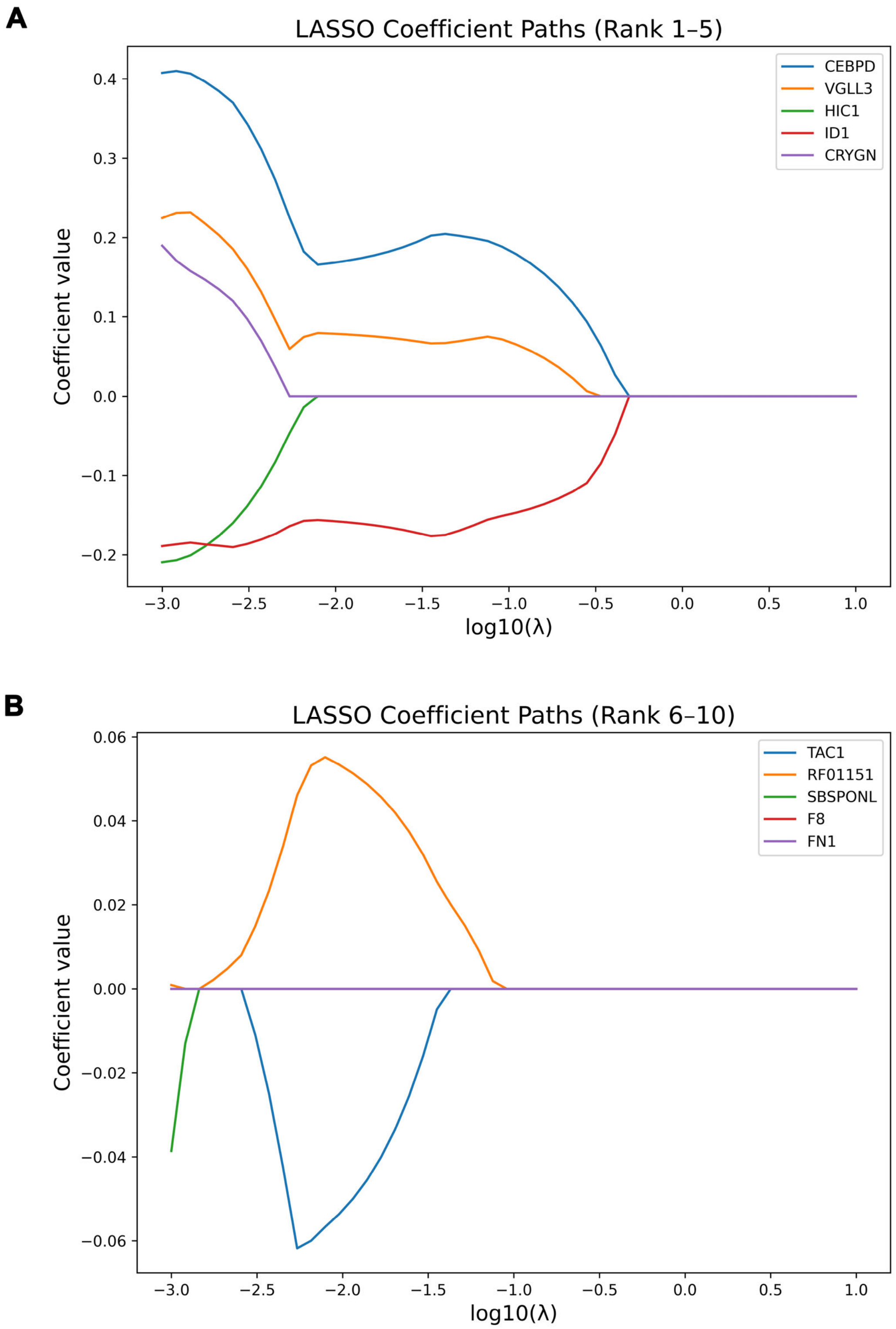

Figure S1. LASSO coefficient paths for genes. Plots illustrate the trajectory of gene coefficients as a function of $\log_{10}(\lambda)$ in the LASSO regression model for (A) top-ranking genes 1–5 and (B) top-ranking genes 6–10. Each line represents a gene, and the vertical line denotes the optimal regularization parameter (λ) selected by cross-validation. Genes ranked below the top 10 had coefficients of zero across all λ values and are therefore not shown.

Table S1. Model training parameters and hyperparameter values. Summary of machine learning hyperparameters used for each classifier. Includes regularization strength (LASSO), kernel and C-value (SVM), and number of estimators and tree depth (RF). Cross-validation scheme and stratification parameters are also reported.

Model	Hyperparameter	Value/Setting
LASSO	Regularization type	L1 penalty (LASSO)
	Solver	saga
	Cross-validation folds	3
	Max iterations	10^{-3} to 10^{3} (log-spaced)
SVM	Kernel	Linear
	Regularization parameter (C)	1
	Penalty	L2
	Max iterations	5000
RBF-SVM	Kernel	Radial basis function
	Regularization parameter (C)	1
	Gamma	0.02
	Calibration method	Sigmoid (3-fold CV)
Random Forest	Number of trees	200
	Maximum tree depth	8
	Minimum sample per leaf	2
	Maximum features per split	0.5

Table S2. Primer sequences for RT-PCR. Primer sequences used for quantitative real-time PCR (qRT-PCR) analysis of candidate genes and controls. Forward (sense) and reverse (antisense) primer sequences are listed in the 5′-to-3′ direction for each target mRNA, including genes associated with wound healing, fibrosis, and housekeeping control (GAPDH) and fibrosis marker (ACTA2).

MRNA	Sense Primer	Antisense Primer
MXRA7	5′- CCATAGGTCTCAGTCACATGAA-3	5′- TGGCTTCCTTCAGCTATCACA-3′
VGLL3	5′- GGAGACCTCTTCTCGATGCG-3′	5′- GCAGCTAATTTCAGGCTCCG-3′
CEBPD	5′- ACTTCTACGACGCCAAGGTG-3′	5′- TAGGAGCTGAAGTCGATGGC-3′
ID1	5′- CTGCAGCACGTGATCGACTA-3′	5′- TCTCTCTCAGCGGCACAGTA-3′
ALDH1A1	5′- CAGACCTTAGGGCAGAGTGC-3′	5′- GGGTGAGCCTTGCTTCTTCA-3′
HS3ST2	5′- CAGAGCAGTGTGTCCGTCAG-3′	5′- ACATGCTGAACAAGGACGCT-3′
LMNA	5′- GTTCACTCTGAAGGCGGGTC-3′	5′- CCTCCTCTCCGTTGGAGTTG-3′
GAPDH	5′- ATTTGGCCGTATTGGCCGCC-3′	5′- AGTGCCCTTGAAGTGTCCGTGT -3′
ACTA2	5′- TGGGCGTACTACAGGGATTG-3′	5′- CGCTCAGGATCTTCATGAGGTAG -3′
